# Concurrent validity of the Gyko inertial sensor system for the assessment of vertical jump height in female sub-elite youth soccer players

**DOI:** 10.1186/s13102-016-0061-x

**Published:** 2016-11-11

**Authors:** Melanie Lesinski, Thomas Muehlbauer, Urs Granacher

**Affiliations:** Division of Training and Movement Science, Research Focus Cognition Sciences, University of Potsdam, Am Neuen Palais 10 (Bldg. 12), 14469 Potsdam, Germany

**Keywords:** Countermovement jump, Squat jump, Accelerometer, Lower-extremity muscle power, Athlete testing, Field test

## Abstract

**Background:**

The aim of the present study was to verify concurrent validity of the Gyko inertial sensor system for the assessment of vertical jump height.

**Methods:**

Nineteen female sub-elite youth soccer players (mean age: 14.7 ± 0.6 years) performed three trials of countermovement (CMJ) and squat jumps (SJ), respectively. Maximal vertical jump height was simultaneously quantified with the Gyko system, a Kistler force-plate (i.e., gold standard), and another criterion device that is frequently used in the field, the Optojump system.

**Results:**

Compared to the force-plate, the Gyko system determined significant systematic bias for mean CMJ (−0.66 cm, *p* < 0.01, *d* = 1.41) and mean SJ (−0.91 cm, *p* < 0.01, *d* = 1.69) height. Random bias was ± 3.2 cm for CMJ and ± 4.0 cm for SJ height and intraclass correlation coefficients (ICCs) were “excellent” (ICC = 0.87 for CMJ and 0.81 for SJ). Compared to the Optojump device, the Gyko system detected a significant systematic bias for mean CMJ (0.55 cm, *p* < 0.05, *d* = 0.94) but not for mean SJ (0.39 cm) height. Random bias was ± 3.3 cm for CMJ and ± 4.2 cm for SJ height and ICC values were “excellent” (ICC = 0.86 for CMJ and 0.82 for SJ).

**Conclusion:**

Consequently, apparatus specific regression equations were provided to estimate true vertical jump height for the Kistler force-plate and the Optojump device from Gyko-derived data. Our findings indicate that the Gyko system cannot be used interchangeably with a Kistler force-plate and the Optojump device in trained individuals. It is suggested that practitioners apply the correction equations to estimate vertical jump height for the force-plate and the Optojump system from Gyko-derived data.

## Background

In many sports (e.g., basketball, handball, soccer, volleyball), vertical jump performance represents an important prerequisite for optimal performance during competition. Therefore, vertical jump tests are frequently used for the assessment of athletic performance, for the evaluation of training-related performance changes over time, and for talent identification [1–3].

The gold standard for the assessment of vertical jump height is the application of force-plates. Compared to force-plates which are expensive and often immobile, inertial sensor systems such as the Gyko device are less expensive, mobile, and thus easy to administer in the field. Further, such a system allows the assessment of jump performance on any surface (e.g., firm ground, sand or grass). Despite these advantages, validity of such devices is an important prerequisite to implement this assessment tool in the field. In this regard, a study of Castagna et al. [4] proved concurrent validity of the Myotest inertial sensor system (Myotest SA, Sion, Switzerland) for the assessment of countermovement jump (CMJ) flight time compared to a Kistler force-plate (i.e., gold standard and criterion device) in trained individuals (i.e., male rugby players aged 16 ± 1 years). They found that validity of the Myotest system is excellent as indicated by an intraclass correlation coefficient (ICC) of 0.88. Further, Requena et al. [5] investigated concurrent validity of the Keimove^TM^ inertial sensor system (Vincid Research, S.L., Granada, Spain) for the assessment of CMJ flight time compared to a IsoNet force-plate (i.e., criterion device) in professional male soccer players (18 ± 3 years). They also revealed excellent ICC values ranging from 0.92 to 0.97. Lastly, Picerno et al. [6] examined the concurrent validity of the inertial sensor system Sensorize (Rome, Italy) for the assessment of CMJ flight time compared to a Bertec force-plate (i.e., criterion device) in male and female college students (25 ± 2 years). Again, these authors reported excellent agreement (ICC = 0.83). However, the Gyko inertial sensor system (Microgate, Bolzano, Italy) contains the latest generation components (i.e., three-dimensional accelerometer, gyroscope, and magnetometer) that provide more accurate and repeatable data of acceleration, angular velocity, and magnetic field in three dimensions. Because of differences in the hard- and software, validity of one inertial sensor systems to another is not transferable.

Therefore, the aim of the present study was to test concurrent (criterion-related) validity of the Gyko inertial sensor system for the assessment of CMJ and squat jump (SJ) height compared to a Kistler force-plate (i.e., gold standard and criterion device) and another criterion device that is frequently used in the field, the Optojump photoelectric cell system. With reference to findings from previous studies [4–7] investigating concurrent validity of inertial sensors for the assessment of jump height, we hypothesized that the Gyko system is a valid tool for estimating vertical jump height obtained during the performance of CMJ and SJ in trained individuals.

## Methods

### Experimental approach to the problem

A single-group study design was used to examine concurrent (criterion-related) validity of the Gyko inertial sensor system for the assessment of CMJ and SJ height. Maximal vertical jump height (dependent variable) was simultaneously assessed using three different test apparatus, the Gyko inertial sensor system, a Kistler force-plate, and Optojump photoelectric cell system in female sub-elite youth soccer players. The Kistler force-plate represents the gold-standard and was used as criterion device. The Optojump system was used as another criterion device because it is frequently used by practitioners in the field. This is due to certain advantages of the Optojump system as compared to force-plates like less expensive, mobile, easy to administer, and applicable on different surfaces (e.g., firm ground, sand).

### Subjects

Nineteen female sub-elite youth soccer players with a mean (±SD) age of 14.7 ± 0.6 years, body height of 165.5 ± 5.4 cm, body mass of 57.6 ± 6.2 kg, and body mass index of 21.0 ± 2.1 kg/m^2^ volunteered to participate in this study. The mean training duration per week amounted to 17.0 h including general and sport-specific conditioning programs. Participants were excluded if they had any history of musculoskeletal, neurological, or orthopaedic disorder in the lower extremities within the preceding six months that might have affected their ability to execute the experimental protocol. Before the start of the study, written informed consent was obtained from the participants and their legal representatives. Ethical permission was given by the ethics committee of the University of Potsdam (submission No. 26/2014) and all experiments were conducted according to the latest version of the declaration of Helsinki.

### Procedures

**Fig. 1:**
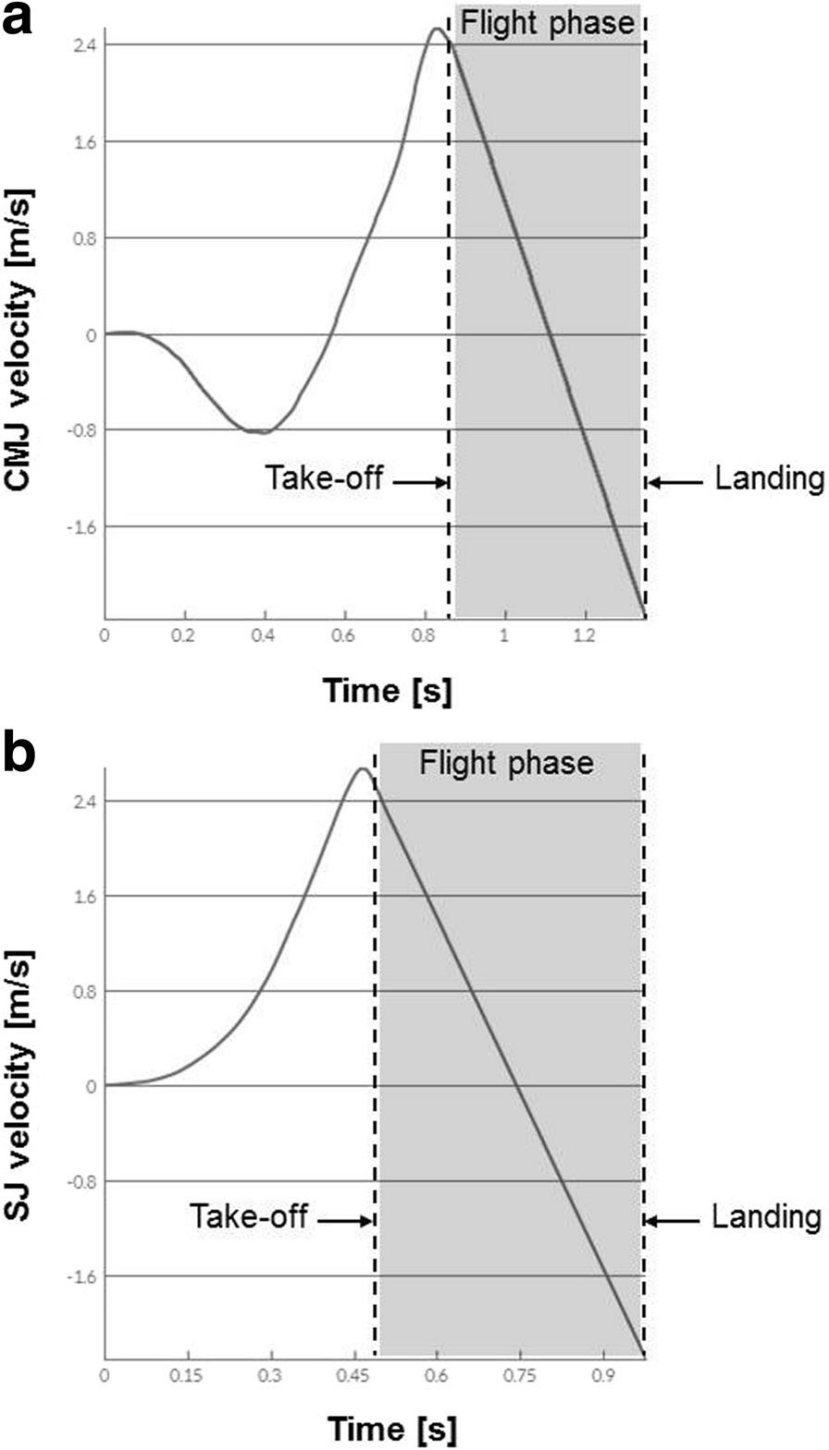
Examples of (**a**) countermovement jump (CMJ) and (**b**) squat jump (SJ) data from an individual recorded by the Gyko inertial sensor system

Prior to testing, all participants underwent a standardized five minute warm-up consisting of submaximal plyometric and skipping exercises. Thereafter, participants were familiarized with the test procedures and performed four jump trials (i.e., one practice and three test trials) without supporting arm swing for each jump type (CMJ, SJ). The rest period between jump trials amounted to 30 s, while a one minute rest was allowed between jump types. During each trial, maximal vertical jump height (dependent variable) was simultaneously assessed using the three different test apparatus (i.e., Gyko inertial sensor system, Kistler force-plate, Optojump photoelectric cell system). Quality of the jump technique was controlled through visual on-site inspection of the experimenter (ML). For the CMJ, participants started from an upright standing position. Subjects were instructed to begin the jump with a downward movement, which was immediately followed by a concentric upward movement, resulting in a maximal vertical jump. During jumping, hands were held akimbo and the depth of the downward movement was freely chosen to allow a natural movement. For the SJ, participants were instructed to start the trial at a 90° knee angle with hands placed on hips. On the start signal, participants had to perform a maximal vertical jump without prior downward movement. All jumps were performed barefoot to avoid bias from shoe-surface interactions. Examples of jump data from an individual recorded by the Gyko inertial sensor system are provided in Fig. 1a and b for the CMJ and the SJ, respectively.

#### Gyko system

**Fig. 2:**
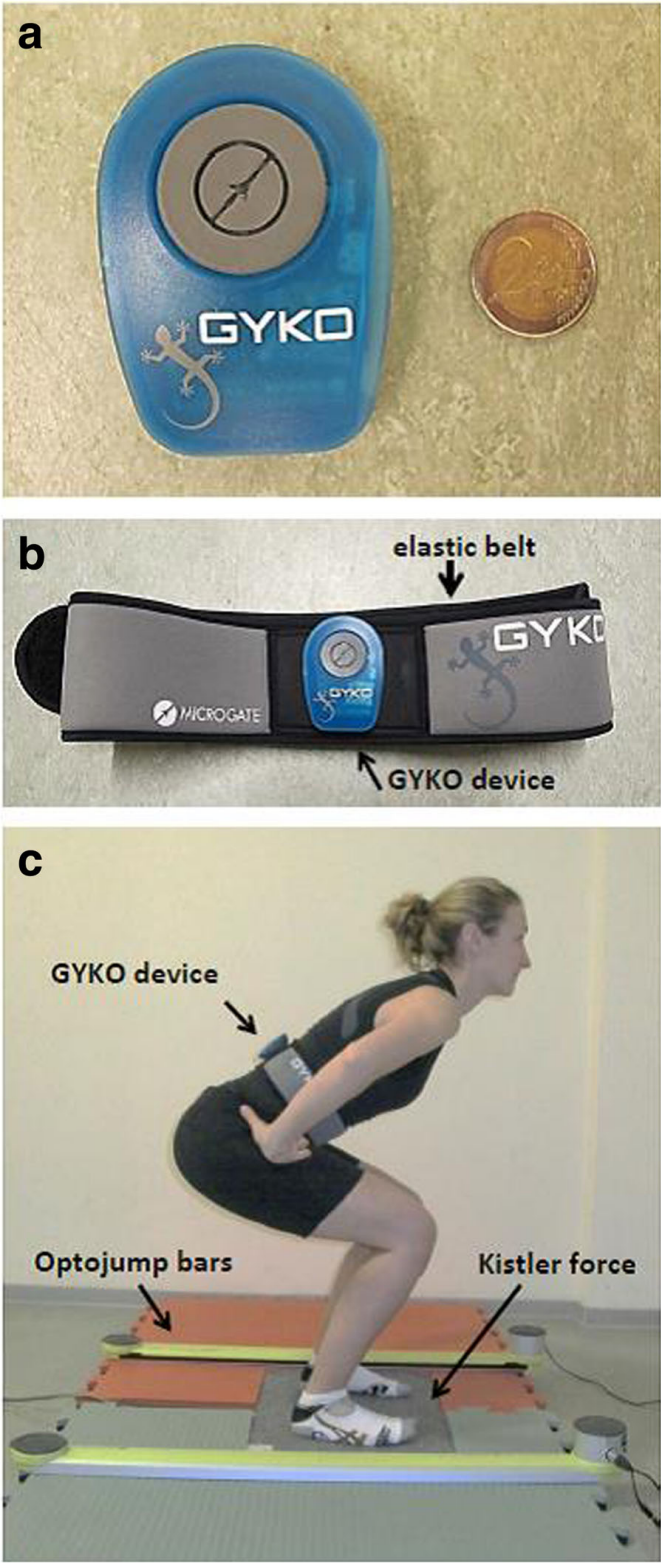
The Gyko inertial sensor system (Microgate, Bolzano, Italy) presented beside a 2 Euro coin (**a**), attached to the proper Velcro elastic belt (**b**), and fixed on the waist level on the back of the body for acceleration recordings during squat jump (**c**)

The Gyko inertial sensor system (dimensions: 50 × 70 × 20 mm, mass: 35 g; Microgate, Bolzano, Italy) contains three-dimensional accelerometer, gyroscope, and magnetometer, which allows recordings (full scale range: 8 g) at a sampling frequency of 500 Hz (Fig. 2a-c). The Gyko system was perpendicularly attached to an elastic belt provided with the system. The Gyko system was fixed on the waist level on the back of the body, as indicated by the manufacturer (http://www.gyko.it/en). During assessment, accelerometer and gyroscope signals are transferred via blue tooth to a personal computer (Lenovo, model T 530) and stored using the proprietary software (GykoRePower Software). The software automatically calculated vertical jump height from the obtained flight time using the following formula: jump height = 1/8 × *g* × *t* ^2^, where *g* is the acceleration due to gravity and *t* is the flight time [8].

#### Kistler force-plate

A three-dimensional Kistler force-plate (dimensions: 600 × 400 × 35 mm; type 9286AA; Kistler, Winterthur, Switzerland) was used as gold standard and criterion device (Fig. 2c). The force-plate was firmly positioned on the ground to measure vertical reaction forces (range: 0–10 kN; sampling rate: 1,000 Hz, FIR-Savitzky Golay Filter) during CMJ and SJ. The force-plate was connected to a personal computer (Sony Vaio, model PCG-51113 M), and the proprietary software (BioWare, type 2812A) allowed jump height quantification from flight time measurements using the aforementioned formula.

#### Optojump device

The Optojump photoelectric cell system (Microgate, Bolzano, Italy) was additionally used as a criterion device and consists of two parallel bars (a transmitter unit and a receiver). The bars were placed approximately one meter apart and parallel to each other (Fig. 2c). The transmitter contains 96 light emitting diodes, which were positioned 0.3 cm from ground level (i.e., vertical distance) at 1.04-cm intervals (i.e., horizontal distance). The Optojump device also measured the flight time of CMJ and SJ with an accuracy of 1,000 Hz. The Optojump system was connected (via USB) to a personal computer (Lenovo, model T 530). Optojump Next software (software version V1.10.7.0) was used for quantification of jump height from flight time measurements. Compared with a force-plate (Kistler, type Quattro Jump), the Optojump device demonstrated excellent concurrent validity (ICC = 0.99) and excellent test-retest reliability (ICC = 0.98) for the estimation of vertical jump height [9]. Thus, we focused our study on the examination of concurrent validity of the Gyko system for the assessment of CMJ and SJ height in comparison to the Kistler force-plate (i.e., gold standard and criterion device) and the Optojump device (another criterion device).

### Statistical analyses

All three test trials of each participant were used for the assessment of concurrent validity. Thus, each jump trial was entered in the calculation as a single case. Data were unimodally distributed and thus presented as mean values and standard deviations (±SD). Validity of test devices were quantitatively assessed with an analysis of variance with repeated measures on the test device (Bonferroni post hoc test), the standard error of measurement (SEM), the intraclass correlation coefficient (ICC; 2.1) and their respective 95 % confidence interval (CI). According to Fleiss’ classification [10], ICC’s > 0.75 indicate “excellent”, between 0.40 and 0.75 “fair to good”, and < 0.40 “poor” relationships. Further, systematic (i.e., group difference) and random (i.e., inter-/intra-individual differences) bias were calculated [11]. Lastly, Bland-Altman plots [12] were provided to identify the magnitude of agreement between devices. For the latter, the differences between Gyko system and Kistler force-plate as well as Optojump device derived values were plotted against the mean of the respective measurements. It is recommended that 95 % of the data points lie within the mean ± 1.96 SDs of the differences between devices. Finally, apparatus specific regression equations were calculated for the estimation of vertical jump height as assessed by the Kistler force-plate and the Optojump device from Gyko-derived data. All analyses were performed using Statistical Package for Social Sciences (IBM® SPSS® Statistics 23). Statistical significance was set at *p* < 0.05.

## Results

**Fig. 3:**
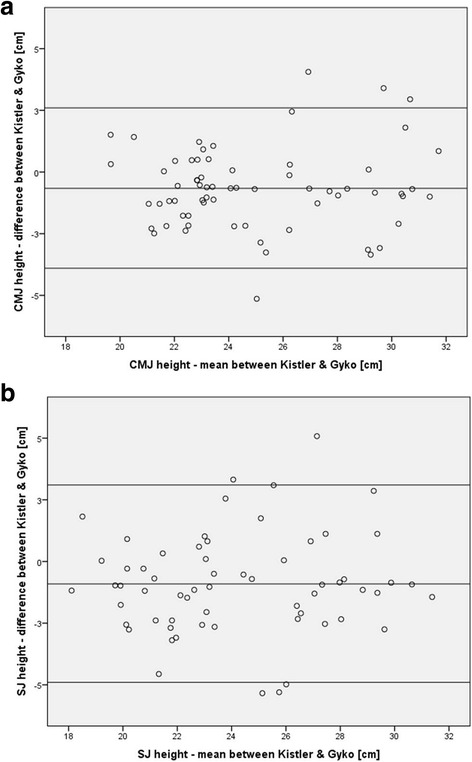
Bland-Altman plot comparing Kistler force-plate and Gyko system derived vertical jump heights (cm) for (**a**) the countermovement jump (CMJ) and (**b**) the squat jump (SJ). The inner line indicates the average of the differences (i.e., systematic bias). The outer lines indicate the limits of agreements corresponding to the mean ± 1.96 SD (i.e., random bias)

Statistical data for the assessment of concurrent validity of the Gyko system as compared to the Kistler force-plate and the Optojump device are presented in Table 1. Compared to the Kistler force-plate, our Gyko analyses revealed significant systematic bias for mean CMJ (−0.66 cm, *p* < 0.01, *d* = 1.41) and mean SJ (−0.91 cm, *p* < 0.01, *d* = 1.69) height. The respective mean vertical jump heights amounted to 24.7 ± 3.5 cm (CMJ) and 23.7 ± 3.6 cm (SJ) for the Gyko system and to 25.3 ± 3.4 cm (CMJ) and 24.7 ± 3.5 cm (SJ) for the Kistler force-plate. Further, random bias was detected for CMJ (±3.2 cm) and SJ (±4.0 cm) height. Additionally, ICCs were “excellent” for CMJ (ICC = 0.87, 95 % CI = 0.77-0.92) and SJ (ICC = 0.81, 95 % CI = 0.66-0.89) height. Lastly, the SEM amounted to 0.59 cm (CMJ) and 0.89 cm (SJ). Figs. 3a-b illustrate Bland-Altman plots for vertical jump height (CMJ and SJ) as assessed by the two apparatus. The figures indicate that 4/66 (6.1 %) and 5/62 (8.1 %) of the data points were beyond the mean ± 1.96 SD lines for CMJ and SJ, respectively. Regression equations were computed to estimate true (Kistler force-plate) vertical jump height using data from the Gyko device. The following equations can be applied: Kistler force-plate CMJ height = 0.858 * Gyko CMJ height + 4.164 cm and Kistler force-plate SJ height = 0.803 * Gyko SJ height + 5.592 cm. Bootstrapping analyses revealed standard errors ranging from 0.064 to 0.067 for the slope and from 1.511 to 1.550 for the intercept of the computed regression equations to estimate true (Kistler force-plate) vertical jump height values from Gyko-derived data.

**Table 1:** Concurrent validity of the Gyko inertial sensor system for the assessment of vertical jump height compared to a Kistler force-plate and the Optojump device

	Gyko vs. Kistler force-plate	Gyko vs. Optojump
Countermovement Jump		
ANOVA	*p* = 0.002 (*d* = 1.41)	*p* = 0.041 (*d* = 0.94)
ICC; 2.1 (95 % CI)	0.87 (0.77–0.92)	0.86 (0.77–0.91)
systematic bias [cm]	−0.66	0.55
random bias (95 % CI) [cm]	±3.2 (−3.9–2.6)	±3.3 (−2.8–3.9)
SEM [cm]	0.59	0.64
Squat jump		
ANOVA	*p* = 0.001 (*d* = 1.69)	*p* = 0.163 (*d* = 0.33)
ICC; 2.1 (95 % CI)	0.81 (0.66–0.89)	0.82 (0.72–0.89)
systematic bias [cm]	−0.91	0.39
random bias (95 % CI) [cm]	±4.0 (−4.9–3.1)	±4.2 (−3.8–4.6)
SEM [cm]	0.89	0.91

ANOVA = Analysis of Variance (Bonferroni post hoc test) and effect size (i.e., Cohen’s *d*) in brackets; ICC; 2.1 = Intraclass Correlation Coefficient; SEM = Standard Error of Measurement; 95 % CI = 95 % Confidence Interval

**Fig. 4:**
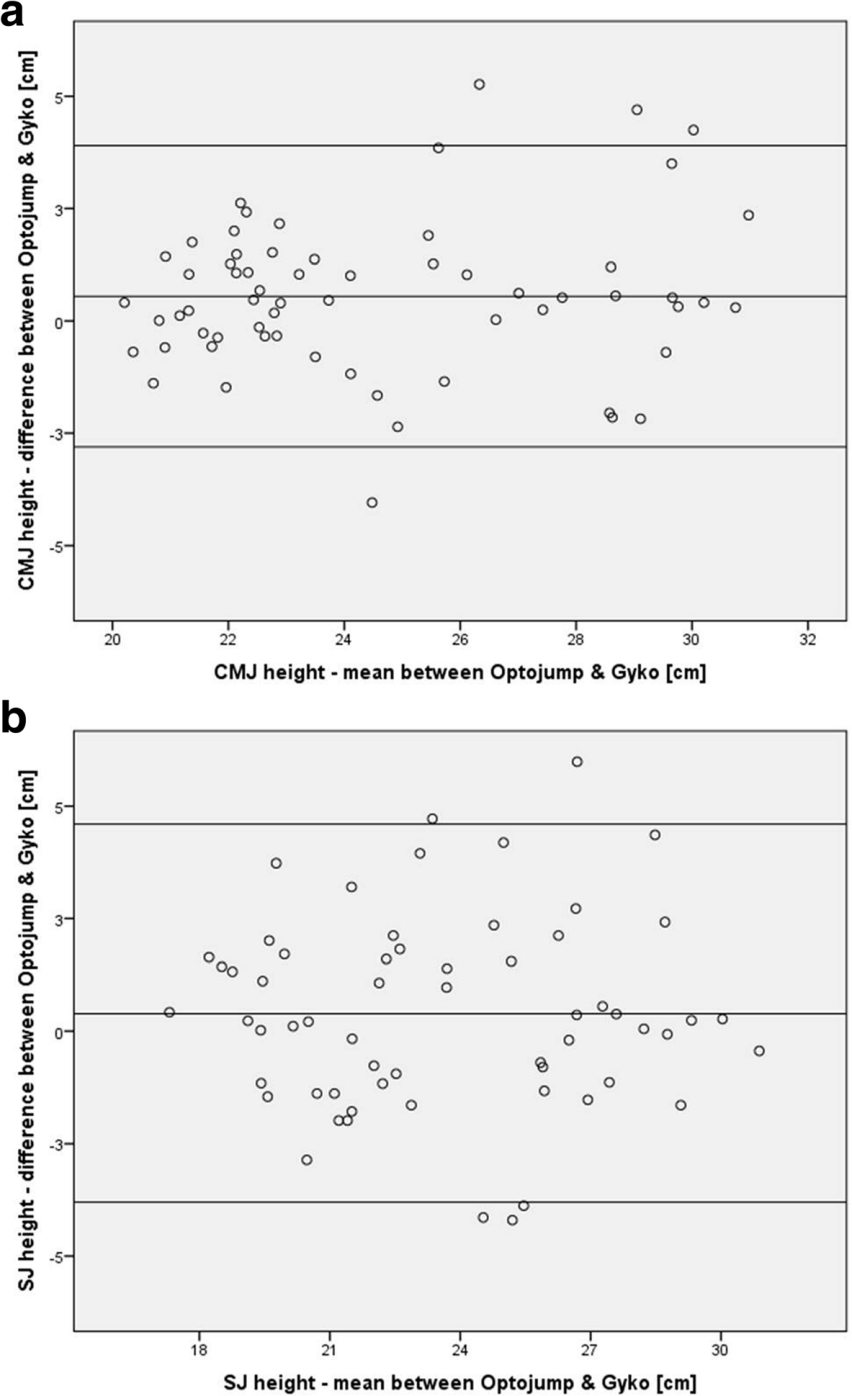
Bland-Altman plot comparing Optojump device and Gyko system derived vertical jump heights (cm) for (**a**) the countermovement jump (CMJ) and (**b**) the squat jump (SJ). The inner line indicates the average of the differences (i.e., systematic bias). The outer lines indicate the limits of agreements corresponding to the mean ± 1.96 SD (i.e., random bias)

Compared to the Optojump device, our Gyko analyses indicated a significant systematic bias for mean CMJ (0.55 cm, *p* < 0.05, *d* = 0.94) but not for mean SJ (0.39 cm) height (Table 1). For the Optojump device, we detected mean vertical jump heights of 24.3 ± 3.2 cm and 23.4 ± 3.6 cm for CMJ and SJ, respectively. Random bias amounted to ± 3.3 cm for CMJ and ± 4.2 cm for SJ height. In addition, ICC values were classified as “excellent” for CMJ (ICC = 0.86, 95 % CI = 0.77-0.91) and SJ (ICC = 0.82, 95 % CI = 0.72-0.89) height. Moreover, the SEM amounted to 0.64 cm (CMJ) and 0.91 cm (SJ). The Bland-Altman plots regarding CMJ and SJ height assessed with the two apparatus are illustrated in Figs. 4a-b, respectively. The charts indicate that 4/63 (6.3 %) and 5/60 (8.3 %) of the data points were beyond the mean ± 1.96 SD lines for CMJ and SJ, respectively. Again, regression equations were computed to estimate true (Optojump) vertical jump height using data from the Gyko device. The following equations can be applied: Optojump CMJ height = 0.815 * Gyko CMJ height + 4.055 cm and Optojump SJ height = 0.820 * Gyko SJ height + 3.891 cm. Bootstrapping analyses revealed standard errors ranging from 0.063 to 0.068 for the slope and from 1.493 to 1.588 for the intercept of the computed regression equations to estimate true (Optojump) vertical jump height values from Gyko-derived data.

## Discussion

The present study simultaneously examined concurrent validity of the Gyko inertial sensor system for the assessment of vertical jump height in female sub-elite youth soccer players compared to a Kistler force-plate (i.e., gold standard and criterion device) and another criterion device that is frequently used in the field, the Optojump system. We assumed that the Gyko system is a valid tool for the estimation of CMJ and SJ height. As a result, we were able to partly confirm our hypothesis which will be discussed in the following.

We detected excellent agreements (ICC = 0.81-0.87) for CMJ and SJ height but significant systematic bias for mean CMJ and mean SJ height between the Gyko system and the Kistler force-plate (i.e., underestimation of Gyko-derived jump height, *p* < 0.01). Our results were in line with those reported in the literature [4–6]. In fact, Castagna et al. [4] examined the concurrent validity of the Myotest inertial sensor system for the assessment of CMJ flight time compared to a Kistler force-plate (i.e., gold standard and criterion device) and the Optojump system (another criterion device) in male rugby players aged 16 ± 1 years. These authors also reported excellent agreement (ICC = 0.88) for CMJ flight time but significant systematic bias (*p* < 0.001) between the two systems (i.e., Kistler force-plate versus Myotest system). In addition, concurrent validity of the Keimove^TM^ inertial sensor system for the assessment of CMJ flight time compared to a IsoNet force-plate (i.e., criterion device) was examined in professional male soccer players (18 ± 3 years) [5]. These authors stated excellent ICC values in the range of 0.92-0.97. Moreover, Picerno et al. [6] compared the Sensorize inertial sensor system during CMJ analyses with data obtained from a Bertec force-plate (i.e., criterion device) in male and female college students (25 ± 2 years). They also found excellent agreement (ICC = 0.83) for CMJ flight time. In terms of systematic bias, the latter two studies [5, 6] showed inconsistent findings. More specifically, Requena et al. [5] found no significant differences, whereas Picerno et al. [6] revealed significant differences (*p* < 0.0001) between the two devices. Further, we found high random bias for CMJ (±3.2 cm) and SJ (±4.0 cm) height between the two devices. The fixation of the Gyko system to an elastic belt produced movement artifacts which could be responsible for the high inter-/intra-individual differences in jump height between the Gyko system and the Kistler force-plate. This could subsequently affect the acceleration-based determination of the exact time at take-off and landing and thus, it may distort flight time and the estimated vertical jump height.

In summary, our findings of excellent agreement but significant systematic and high random bias indicate that the Gyko system cannot be used interchangeably with the Kistler force-plate in trained individuals. Consequently, apparatus specific regression equations were provided to estimate CMJ and SJ height for the force-plate from Gyko-derived data. However, these are preliminary equations based on a small sample size, a specific age range, and female sub-elite youth soccer players.

Besides the comparison of Kistler force-plate versus Gyko-derived data, we additionally examined the concurrent validity of the Gyko compared to the Optojump system. As a result, we detected excellent agreement for CMJ (ICC = 0.86) and SJ (ICC = 0.82) heights as well as significant systematic bias for mean CMJ (i.e., overestimation of Gyko-derived jump height, *p* < 0.05) but not for mean SJ heights. In addition, random bias was high for CMJ (±3.3 cm) and SJ (±4.2 cm) height between the two systems. Thus, preliminary regression equations were established for the estimation of true (Optojump) vertical jump height values from Gyko-derived data. Our findings are in line with the available literature [4, 7]. As mentioned before, Castagna et al. [4] compared data from the Myotest system with those obtained from the Optojump device. They reported significant systematic bias (*p* < 0.001) between the two systems as indicated by longer CMJ flight times (corresponds to larger CMJ heights) for the Myotest system. However, these authors did not report the observed agreement (i.e., ICC values) between the two devices. Such correlation analyses were provided by Casartelli et al. [7] who compared the Myotest system during CMJ and SJ analyses with data obtained from the Optojump system in male basketball players (15 ± 4 years). Casartelli et al. [7] found excellent agreement (ICC = 0.98) for CMJ and SJ heights as well as significant systematic bias (i.e., overestimation of Myotest-derived jump height, *p* < 0.001) for mean CMJ and mean SJ height between the two devices. The overestimation of Gyko compared to Optojump-derived CMJ heights might be caused by methodological differences in vertical jump assessment. In fact, the Gyko system (fixed at waist level) starts measuring flight time as predefined body acceleration occurs. Contrary to the Gyko system, the Optojump photoelectric cell system starts measuring flight time at the instant when the toes leave the ground. At this point, communication of the light emitting diodes (0.3 cm above the floor level) between the transmitter and receiver bar is enabled. This may partially explain the observed discrepancy between Gyko and Optojump-derived vertical jump heights. The high random bias between Gyko and Optojump might be due to displacements of the elastic belt which may bias acceleration-based vertical jump height detection.

In general, despite of the preliminary regression equations for the estimation of true (Optojump, Kistler) vertical jump height values from Gyko-derived data, the detected high random bias of the Gyko system (i.e., CMJ height = ~ 3 cm; SJ height = ~ 4 cm) compared to the Kistler force-plate and the Optojump system may hinder the accurate inter-/intra-individual assessment of training-related performance changes over time. For example, Rubley et al. [13] examined the effects of general soccer training plus plyometric exercises (plyometric group) versus general soccer training only (control group) in female adolescent soccer players (13 ± 1 years). Following seven weeks of training, they found an improvement of 3.3 cm for vertical jump height in the plyometric group that is close to the observed random bias in our study.

Two potential limitations of this study warrant discussion. First, the algorithm employed for estimating vertical jump height (i.e., jump height = 1/8 × g × t^2^) could be a potential error source [14]. For example, an approach employing direct integration of vertical acceleration data with subsequent correction based on known kinematics [15] or ballistic motion equations that employ estimates of vertical jump height and vertical velocity at take-off [6], have each provided experimental results with reduced systematic bias and random error. Thus, altering the algorithm for estimating vertical jump height could be a means to improve the reported results. Second, the Gyko device was primarily developed as an add-on instead for standalone use. Thus, Microgate company recommends using the Gyko device together with the Optojump system for the assessment of data (e.g., work, duration) that are related to the eccentric and concentric jump phase.

## Conclusions

The present study examined concurrent validity of vertical jump height using the Gyko inertial sensor system compared to a Kistler force-plate (i.e., gold standard and criterion device), and the Optojump system (another criterion device) in female sub-elite youth soccer players. We detected significant systematic and high random bias for mean CMJ and mean SJ height between the Gyko system, the Kistler force-plate, and the Optojump device. This indicates that the Gyko system cannot be used interchangeably with a Kistler force-plate and the Optojump device for individual comparisons. From a practical perspective, practitioners are advised to apply the calculated apparatus specific regression equations to estimate vertical jump height for the Kistler force-plate and the Optojump device from Gyko-derived data.

## Abbreviations

CI: Confidence interval
CMJ: Countermovement jump
ICC: Intraclass correlation coefficient
SD: Standard deviation
SEM: Standard error of measurement
SJ: Squat jump
